# Proportion of Deaths and Clinical Features in Bundibugyo Ebola Virus Infection, Uganda

**DOI:** 10.3201/eid1612.100627

**Published:** 2010-12

**Authors:** Adam MacNeil, Eileen C. Farnon, Joseph Wamala, Sam Okware, Deborah L. Cannon, Zachary Reed, Jonathan S. Towner, Jordan W. Tappero, Julius Lutwama, Robert Downing, Stuart T. Nichol, Thomas G. Ksiazek, Pierre E. Rollin

**Affiliations:** Author affiliations: Centers for Disease Control and Prevention, Atlanta, Georgia, USA (A. MacNeil, E.C. Farnon, D.L. Cannon, Z. Reed, J.S. Towner, S.T. Nichol, T.G. Ksiazek, P.E. Rollin);; Ministry of Health, Republic of Uganda, Kampala, Uganda (J. Wamala, S. Okware);; Global AIDS Program, Centers for Disease Control and Prevention, Entebbe, Uganda (J.W. Tappero, R. Downing);; Uganda Virus Research Institute, Entebbe (J. Lutwama);; University of Texas Medical Branch, Galveston, Texas, USA (T.G. Ksiazek)

**Keywords:** Ebola, hemorrhagic fever, Ebolavirus, Bundibugyo, outbreak, proportion of deaths, laboratory-confirmed, Uganda, viruses, dispatch, *Suggested citation for this article:* MacNeil A, Farnon EC, Wamala J, Okware S, Cannon DL, Reed Z, et al. Proportion of deaths and clinical features in Bundibugyo Ebola virus infection, Uganda. Emerg Infect Dis [serial on the Internet]. 2010 Dec [*date cited*]. http://dx.doi.org/10.3201/eid1612.100627

## Abstract

The first known Ebola hemorrhagic fever (EHF) outbreak caused by Bundibugyo Ebola virus occurred in Bundibugyo District, Uganda, in 2007. Fifty-six cases of EHF were laboratory confirmed. Although signs and symptoms were largely nonspecific and similar to those of EHF outbreaks caused by Zaire and Sudan Ebola viruses, proportion of deaths among those infected was lower (≈40%).

Ebola hemorrhagic fever (EHF) is a severe disease caused by several species of *Ebolavirus* (EBOV), in the family *Filoviridae.* Before 2007, four species of EBOV had been identified; 2 of these, *Zaire ebolavirus* and *Sudan ebolavirus*, have caused large human outbreaks in Africa, with proportion of deaths ≈80%–90% and 50%, respectively (*1**–**5*). Large outbreaks are associated with person-to-person transmission after the virus is introduced into humans from a zoonotic reservoir. Data suggest that this reservoir may be fruit bats (*6**,**7*). During outbreaks of EHF, the virus is commonly transmitted through direct contact with infected persons or their bodily fluids (*8**–**11*). The onset of EHF is associated with nonspecific signs and symptoms, including fever, myalgias, headache, abdominal pain, nausea, vomiting, and diarrhea; at later stages of disease, overt hemorrhage has been reported in ≈45% of cases (*12*).

Bundibugyo District is located in western Uganda, which borders the Democratic Republic of Congo. After reports of a mysterious illness in Bundibugyo District, the presence of a novel, fifth EBOV virus species, *Bundibugyo ebolavirus* (BEBOV), was identified in diagnostic samples submitted to the Centers for Disease Control and Prevention (CDC), Atlanta, Georgia, USA, in November 2007 (*13*). In response to detection of EBOV, an international outbreak response was initiated. In this report, we summarize findings of laboratory-confirmed cases of BEBOV infection.

## The Study

Anecdotal reports suggested that human illness consistent with a viral hemorrhagic fever arose in Bundibugyo District as early as August 2007. After EHF was confirmed (*13*), isolation wards were established at 2 medical facilities in the district. Diagnostic samples from hospitalized patients with acute illness and community residents who had febrile illnesses and multiple additional signs, symptoms, or epidemiologic exposures suggestive of EHF, were routinely collected for EBOV testing. These signs and symptoms included headache, vomiting, diarrhea, abdominal pain, conjunctivitis, skin rash, muscle pain, fatigue, difficulty swallowing, difficulty breathing, hiccups, bleeding, unexplained death, or contact with a patient with suspected EHF. A laboratory-confirmed case of EHF was defined as illness in a person whose diagnostic samples were found positive for EBOV infection by any of the following tests: PCR (*13*), virus isolation, antigen detection, or immunoglobulin (Ig) M ELISA (*14**,**15*).

In addition, in a subset of surviving persons who had a history of illness consistent with EHF but no acute-phase blood samples available for testing, a convalescent-phase blood sample was collected for laboratory confirmation by IgG ELISA (*14**, **15*). Laboratory testing was performed at the Uganda Viral Research Institute in Entebbe, and subsequent testing was performed on some samples at CDC, Atlanta. This analysis is limited to laboratory-confirmed EHF cases, although additional suspected cases were identified during the outbreak.

Fifty-six confirmed cases of EHF were identified; 43 of these were diagnosed on the basis of positive test results from acute-phase specimens (Figure). Twenty-six patients had a positive EBOV IgG titer in convalescent-phase serum, including 13 persons who had evidence of EBOV infection in acute-phase and convalescent-phase samples.

**Figure:**
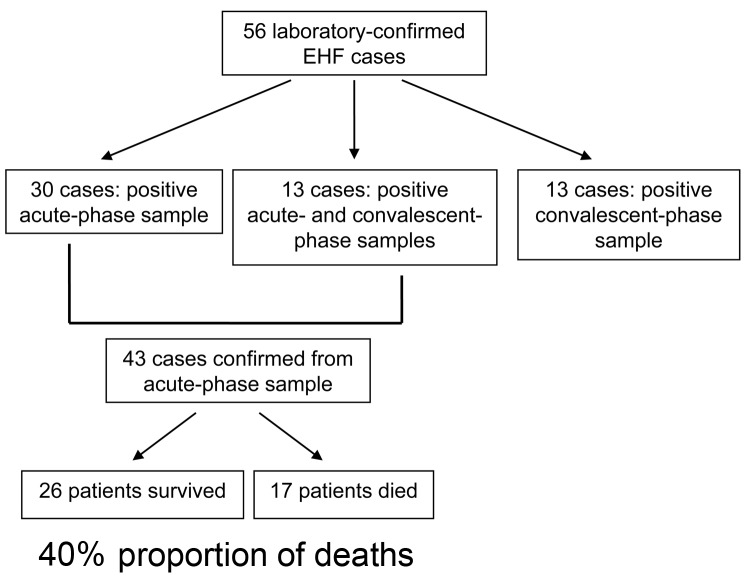
Number of laboratory-confirmed Ebola hemorrhagic fever (EHF) cases diagnosed on the basis of positive acute-phase or convalescent-phase diagnostic samples and calculation of proportion of deaths among case-patients who had an acute-phase diagnostic sample, Bundibugyo District, Uganda, 2007.

The proportion of deaths during this outbreak was calculated for case-patients confirmed on the basis of an acute-phase diagnostic sample (those who only had a convalescent-phase sample are by definition surviving case-patients, and represent a biased sample). Of the 43 cases confirmed from acute-phase samples, 17 deaths occurred, for a proportion of 40%. The mean age of those who died (42 years, range 20–70 years) was significantly higher than that of survivors (33 years, range 12–50 years; p = 0.0390). No gender bias was observed between survivors and those who died (Table).

**Table:** Demographic characteristics and signs and symptoms for 56 case-patients who had laboratory-confirmed EHF, Bundibugyo District, Uganda, in 2007*

Characteristic	Case-patients confirmed by acute-phase sample, n = 43			Total no. confirmed case-patients, n = 56
	No. survived, n = 26	No. died, n = 17	p value	
Mean age, y (range)	33 (12–50)	42(20–70)	0.039†	37.4 (11–70)
Male sex (%)	16 (62)	8 (47)	>0.100‡	30 (54)
Mean incubation period, d (95% CI)§	5.7 (4.4–7.0)	7.4 (5.4–9.3)	>0.100†	6.3 (5.2–7.3)
Signs and symptoms, no. reporting/no. available (%)¶				
Fever	26/26 (100)	16/16 (100)	>0.100	55/55 (100)
Fatigue	22/23 (96)	14/14 (100)	>0.100	49/50 (98)
Headache	21/25 (84)	14/15 (93)	>0.100	48/53 (91)
Nausea/vomiting	24/26 (92)	13/15 (87)	>0.100	48/54 (89)
Abdominal pain	23/26 (88)	13/14 (93)	>0.100	47/53 (89)
Muscle/joint pain	19/23 (83)	12/14 (86)	>0.100	44/50 (88)
Diarrhea	24/26 (92)	13/15 (87)	>0.100	47/54 (87)
Anorexia/weight loss	19/23 (83)	12/15 (80)	>0.100	43/51 (84)
Difficulty swallowing	10/23 (43)	6/15 (60)	>0.100	27/51 (53)
Rash	9/26 (35)	5/15 (33)	>0.100	25/54 (46)
Difficulty breathing	6/23 (26)	8/14 (57)	0.085	23/50 (46)
Hiccups	4/23 (17)	6/15 (40)	>0.100	16/51 (31)
Bleeding#	11/26 (42)	9/17 (53)	>0.100	30/56 (54)

*EHF, Ebola hemorrhagic fever; CI, confidence interval. †By 2-sample t-test. ‡By χ^2^ test. §For case-patients reporting contact with a confirmed case-patient, or contact with case-patient X (see text for full description), based on time from last reported contact with case-patient to development of signs and symptoms (n = 16 for case-patients in Survived column; n = 8 for case-patients in Died column; n = 24 for all confirmed case-patients). ¶No. case-patients reporting the sign or symptom/no. case-patients with information available about presence of that the sign or symptom. p value was determined by Fisher exact test in which a comparison was made between the proportion of case-patients who survived vs. those who died. #At least 1 of the following manifestations: bleeding from injection site, gums, eyes, nose, vagina; black or bloody stool; bloody vomitus; hematuria.

Signs and symptoms were reported by patients on standardized surveillance case-report forms at the time of case identification, and in some instances, were examined further by chart review or follow-up interview. Common symptoms among patients with laboratory-confirmed cases included fever, fatigue, headache, nausea/vomiting, abdominal pain, muscle/joint pain, diarrhea, and anorexia/weight loss (Table). No difference in the proportion of those reporting signs and symptoms, or between those who survived and those who died was noted, except for difficulty swallowing. This symptom was more common among case-patients who died (though marginally significant, p = 0.0851). Bleeding of any type (bleeding from injection site, gums, eyes, nose, vagina; black or bloody stool; bloody vomitus; or hematuria) was reported among 54% of all patients with laboratory-confirmed cases.

As part of the standardized surveillance case-report form, patients were also asked whether they had had contact with a sick person during the 3 weeks before development of illness. A large portion of the laboratory-confirmed case-patients in this outbreak reported direct contact with a specific person, (case X), who died of a severe hemorrhagic febrile illness consistent with EHF (no diagnostic specimens were collected from this person) in November 2007. Using the date of last contact for those reporting contact with case X or reporting contact with another laboratory-confirmed case-patient to the date of symptom onset, we calculated an average incubation period of 6.3 days among laboratory-confirmed EHF case-patients (n = 24). No significant difference was noted in the incubation period between survivors (5.7 days) and those who died (7.4 days).

## Conclusions

The 2007 outbreak of EHF in Bundibugyo District, Uganda, was caused by a new EBOV species, *Bundibugyo ebolavirus* (*13*). Previous outbreaks of EHF have resulted in high proportion of deaths, ranging from 50% to 90% (*1**–**5*). The case fatality proportion of deaths among case-patients with EHF confirmed by acute diagnostic specimens in this outbreak was 40%, a lower percentage than for other species of EBOV that have caused human outbreaks in Africa. However, we cannot exclude the possibility that the lower proportion of deaths in this outbreak is an artifact of differences in the severity of laboratory-confirmed cases detected through outbreak surveillance or the quality of care received by hospitalized laboratory-confirmed EHF case-patients in Bundibugyo District. Nonetheless, sustained person-to-person transmission was sufficient to result in a sizeable outbreak, and death was clearly not uncommon. Thus, BEBOV should be considered a pathogen of serious public health concern.

As with previously documented EHF outbreaks, older age appeared to be a risk factor for death (*3*). The incubation period of EHF was ≈1 week, and signs and symptoms were largely nonspecific; infections frequently involved fever, fatigue, headache, gastrointestinal involvement, and muscle and joint pain. The nonspecific nature of these signs and symptoms, which may mimic other tropical diseases, made diagnosis of EHF based on clinical characteristics alone particularly challenging and underscores the importance of laboratory-based diagnostics to confirm and monitor control and response efforts for EHF outbreaks. Notably, signs and symptoms described in this report are primarily based on information from patient surveillance case-report forms filled out at the time of triage, and they may not represent the full spectrum of illness experienced by all persons with EHF during this outbreak.

It is apparent that novel emerging infections continue to occur. The outbreak of EHF described in this report involved a previously unidentified EBOV species, with a proportion of deaths of 40%. BEBOV represents the fourth EBOV species–associated disease in humans, and the third species to cause large human outbreaks of EHF. Although proportion of deaths was lower than that documented in previous EHF outbreaks, BEBOV is a severe human pathogen with epidemic potential. These findings demonstrate the need for increased surveillance and diagnostic capabilities, as well as the capacity to respond quickly to emerging human infections.
